# Identifying key m^6^A-methylated lncRNAs and genes associated with neural tube defects *via* integrative MeRIP and RNA sequencing analyses

**DOI:** 10.3389/fgene.2022.974357

**Published:** 2022-11-22

**Authors:** Jing Yang, Jing Xu, Luting Zhang, Yingting Li, Min Chen

**Affiliations:** ^1^ Department of Obstetrics, Affiliated Xiaoshan Hospital, Hangzhou Normal University, Hangzhou, Zhejiang, China; ^2^ Department of Obstetrics and Gynecology, The First Affiliated Hospital of Kunming Medical University, Kunming, Yunnan, China; ^3^ Department of Obstetrics and Gynecology, Department of Fetal Medicine and Prenatal Diagnosis, Key Laboratory for Major Obstetric Diseases of Guangdong Province, The Third Affiliated Hospital of Guangzhou Medical University, Guangzhou, Guangdong, China

**Keywords:** neural tube defects, N6-methyladenosine modification, long non-coding RNA, functional enrichment analysis, methylated RNA immunoprecipitation sequencing

## Abstract

**Objective:** N^6^-methyladenosine (m^6^A) is a common post-transcriptional modification of messenger RNAs (mRNAs) and long non-coding RNAs (lncRNAs). However, m^6^A-modified lncRNAs are still largely unexplored. This study aimed to investigate differentially m^6^A-modified lncRNAs and genes involved in neural tube defect (NTD) development.

**Methods:** Pregnant Kunming mice (9–10 weeks of age) were treated with retinoic acid to construct NTD models. m^6^A levels and methyltransferase-like 3 (*METTL3*) expression were evaluated in brain tissues of the NTD models. Methylated RNA immunoprecipitation sequencing (MeRIP-seq) and RNA sequencing (RNA-seq) were performed on the NovaSeq platform and Illumina HiSeq 2,500 platform, respectively. Differentially m^6^A-methylated differentially expressed lncRNAs (DElncRNAs) and differentially expressed genes (DEGs) were identified, followed by GO biological process and KEGG pathway functional enrichment analyses. Expression levels of several DElncRNAs and DEGs were evaluated by quantitative reverse transcription-polymerase chain reaction (qRT-PCR) for validation.

**Results:** m^6^A levels and *METTL3* expression levels were significantly lower in the brain tissues of the NTD mouse model than in controls. By integrating MeRIP-seq and RNA-seq data, 13 differentially m^6^A-methylated DElncRNAs and 170 differentially m^6^A-methylated DEGs were identified. They were significantly enriched in the Hippo signaling pathway and mannose-type *O*-glycan biosynthesis. The qRT-PCR results confirmed the decreased expression levels of lncRNAs, such as Mir100hg, Gm19265, Gm10544, and Malat1, and genes, such as *Zfp236*, *Erc2*, and *Hmg20a*, in the NTD group.

**Conclusion:** *METTL3*-mediated m^6^A modifications may be involved in NTD development. In particular, decreased expression levels of Mir100hg, Gm19265, Gm10544, Malat1, *Zfp236*, *Erc2*, and *Hmg20a* may contribute to the development of NTD.

## Introduction

Neural tube defects (NTDs) are common congenital abnormalities caused by the failure of the neural tube to close during embryogenesis (Yadav et al., 2021). The prevalence of NTDs is estimated to be 18.6 per 10,000 live births (Finnell et al., 2021). Babies with NTDs are more likely to be stillborn, die shortly after birth, or develop different degrees of disability (Huang et al., 2021). The etiology of NTDs is complex and is associated with interactions between genetic factors and diverse environmental factors (Avagliano et al., 2019; Kakebeen and Niswander, 2021). However, the molecular mechanisms underlying NTDs have not yet been fully elucidated.

An N^6^-methyladenosine (m^6^A) modification is a dynamic and reversible process modulated by methyltransferase “writers” (such as methyltransferase-like 3 (*METTL3*)) and demethylase “erasers” (such as alkB homolog 5 (*ALKBH5*)) (Zhang W. et al., 2021). m^6^A modifications are crucial for the regulation of RNA metabolism, including RNA stability, translation, alternative splicing, and translocation (Jiang et al., 2021). Moreover, m^6^A modifications have functions in embryonic development and neurodevelopmental diseases (Yen and Chen, 2021). m^6^A is the most prevalent messenger RNA (mRNA) and long non-coding RNA (lncRNA) modification (Tang et al., 2021). lncRNAs are a group of RNA transcripts longer than 200 nucleotides without open reading frames (Iyer et al., 2015). They have been implicated in the development of neurodevelopmental and neuropsychiatric disorders (Aliperti et al., 2021). Furthermore, m^6^A-modified lncRNAs are involved in various diseases. For instance, the lncRNA DNA methylation-deregulated and RNA m6A reader-cooperating lncRNA (*DMDRMR*) interacts with the m^6^A reader insulin-like growth factor 2 mRNA-binding protein 3 (IGF2BP3) to stabilize target genes, like cyclin-dependent kinase 4 (*CDK4*), in an m^6^A-dependent manner, thus exerting an oncogenic effect in clear cell renal cell carcinoma (Gu et al., 2021). m^6^A modifications of the lncRNA ZNFX1 Antisense RNA 1 (*ZFAS1*) and *RAB22A*, member RAS oncogene family (*RAB22A*) *via* METTL14 contributes to the development of atherosclerosis (Gong et al., 2021). Additionally, m^6^A-modified lncRNAs play pivotal roles in obstructive nephropathy (Liu P. et al., 2020), intervertebral disc degeneration (Li et al., 2022), and muscle development (Xie et al., 2021). However, the key m^6^A-modified lncRNAs and their regulatory mechanisms in NTD development have not yet been thoroughly investigated.

In the present study, we constructed a mouse model of NTD and investigated the overall m^6^A levels and *METTL3* expression. We then performed m^6^A-modified RNA immunoprecipitation sequencing (MeRIP-seq) and RNA sequencing (RNA-seq) to compare brain tissues of NTD embryos and control embryos and conducted comprehensive bioinformatics analyses to identify key m^6^A-modified lncRNAs and genes associated with NTDs. Moreover, the expression levels of m^6^A-modified lncRNAs and genes were experimentally validated. These results are expected to improve our understanding of the molecular mechanisms underlying NTDs.

## Materials and methods

### Animal models and samples

The Ethics Committee of the Third Affiliated Hospital of Guangzhou Medical University approved this study (2022-041, date of approval: 1 June 2022). Equal numbers of male and female Kunming mice (9–10 weeks) (Caven Biogle (Suzhou) Model Animal Research Co. Ltd., Suzhou, China) were mated overnight. The vaginal plug was examined the following day, and 25 pregnant mice were used for subsequent experiments. The day (08:00) a vaginal plug was observed was regarded as the embryonic day 0 (E0d), and 16:00 was considered E0.5d. NTD models were established as described previously (Yu et al., 2017). On E7.0d–7.25d, the mice in the NTD group (N = 18) were administered corn oil-dissolved retinoic acid (50 mg/kg of body weight) (R2625; Sigma, St. Louis, MO, USA) by one-time gavage. Mice in the control group (N = 7) were administered an equal amount of corn oil. On E16.5d, the mice were sacrificed by cervical dislocation, and embryos were taken from the uteri. NTDs were confirmed using a dissecting microscope. Brain tissues (anterior end of the neural tube) of NTD and control embryos were collected and frozen for storage.

### m^6^A quantification

Total RNA was isolated from the brain tissues of the NTD and control groups using TRIzol reagent (Invitrogen, Carlsbad, CA, USA). Using an m^6^A RNA Methylation Quantification Kit (Abcam, Cambridge, MA, USA), m^6^A levels were colorimetrically quantified by determining absorbance at 450 nm.

### Quantitative reverse transcription-polymerase chain reaction (qRT-PCR)

m^6^A methyltransferase *METTL3* expression in brain tissues of the NTD and control groups was detected by real-time qRT-PCR. Total RNA was extracted from brain tissues using TRIzol reagent (Invitrogen). Reverse transcription for cDNA synthesis was performed using the PrimeScript™ first strand cDNA Synthesis Kit (Takara, Beijing, China). Real-time qRT-PCR was conducted using Power SYBR Green PCR Master Mix (Thermo Fisher Scientific, Waltham, MA, USA) and the 7900HT Fast qPCR System (Applied Biosystems, Foster City, CA, USA). Cycling conditions were as follows: initial denaturation at 95°C for 10 min and 40 cycles of 95°C for 15 s and 60°C for 60 s, followed by a melt curve analysis from 60°C to 95.0°C in increments of 0.5°C per 10 s. The internal control was glyceraldehyde-3-phosphate dehydrogenase (*GAPDH*), and the relative expression levels of lncRNAs and genes were calculated using the 2^−ΔΔCT^ method.

### Methylated RNA immunoprecipitation (IP) sequencing (MeRIP-seq) and differential methylation analysis

Total RNA was extracted from the brain tissues of three NTD embryos and three control embryos using TRIzol reagent (Invitrogen) and was treated with DNase I (Roche Diagnostics, Mannheim, Germany) to remove residual DNA. RNA was fragmented and immunoprecipitated with a mixture of beads and an anti-m^6^A antibody (Abcam) for 6 h at 4°C. The mixture was then immunoprecipitated by incubation with beads resuspended in IP reaction buffer (fragmented RNA, 5′ IP buffer, and RNasin Plus RNase Inhibitor) at 4°C for another 2 h. Then, the immunoprecipitated RNA was eluted and used for m^6^A MeRIP library construction using the SMARTer Stranded Total RNA-Seq Kit v2 (Pico Input Mammalian, Takara/Clontech), following the manufacturer’s protocols. Sequencing was performed on the NovaSeq platform. The raw data have been deposited in the NCBI Sequence Read Archive (SRA) database under accession number PRJNA879256.

Raw reads were filtered using Trimmomatic (v0.36) (Bolger et al., 2014), followed by a quality assessment to ensure the reliability of subsequent analyses. The data were aligned to the reference genome (mm10_gencode) using STAR (v2.5.2a) (Dobin et al., 2013). Uniquely mapped reads were used for subsequent analyses. Peak calling was used to detect regions significantly enriched in RNA (peaks), which were candidate m^6^A-methylated sites. Peak calling was performed using the MetPeak package (Cui et al., 2016) in R (v4.1.0).

Differentially methylated sites on RNAs (m^6^A peaks) between NTD and control samples were analyzed using MeTDiff (Cui et al., 2018) in R based on MeRIP-seq data. The cutoff values were |fold change| > 1 and *p* < 0.05. RNAs with differentially methylated m^6^A sites were classified as mRNAs, lncRNAs, miRNAs, pseudogenes, or others to better understand the function of m^6^A methylation in various pathological processes. Moreover, the R package ChIPseeker (v1.24.0) (Yu et al., 2015) was used to annotate differentially methylated m^6^A sites and to analyze their distribution in functional regions according to their locations in RNA transcripts (i.e., 5′ untranslated region (UTR), 3′UTR, first exon, other exon, first intron, other intron, and distal intergenic regions). mRNAs with differentially methylated m^6^A sites in the 3′UTR region were analyzed.

### RNA sequencing (RNA-seq)

Total RNA was isolated from the brain tissues of five NTD embryos and five control embryos using TRIzol reagent (Invitrogen), following RNA concentration detection using the NanoDrop 2000. Ribosomal RNA (rRNA) was removed, and the RNA was fragmented. The RNA library was established using the Illumina TruSeq RNA Sample Prep Kit and then loaded on an Illumina HiSeq 2,500 platform for 150 bp paired-end sequencing. The raw data have been deposited in the NCBI Sequence Read Archive (SRA) database under accession number PRJNA879256.

Raw reads were subjected to data filtration to remove low-quality reads. Read alignment to the reference genome (Mus_musculus.GRCm39) was conducted using HISAT2 (v2.1.0) (Kim et al., 2015). lncRNA and mRNA read counts were generated using RSEM (Li and Dewey, 2011), and mRNA or lncRNA expression levels were quantified.

To explore the key molecules in the pathogenesis of NTD, differentially expressed genes (DEGs) and differentially expressed lncRNAs (DElncRNAs) between NTD and control samples were identified using the DESeq2 package (v1.22.2) in R based on RNA-seq data. The *p*-value was adjusted using the Benjamini–Hochberg (BH) method (Benjamini and Hochberg, 1995). The cutoff values were adjusted *p* < 0.05 and |fold change| > 1.

### Integrative lncRNA/mRNA analysis with differentially methylated m^6^A sites and DElncRNAs/DEGs

Data from MeRIP-seq and RNA-seq analyses were integrated. Then, m^6^A-methylated DElncRNAs and DEGs in which m^6^A levels were negatively correlated with expression levels were identified by analyzing the m^6^A levels of lncRNAs/mRNAs with differentially methylated m^6^A sites and DElncRNA/DEG expression levels.

### Construction of a lncRNA-mRNA co-expression network

The “cor” function in R was used to calculate the Pearson correlation coefficients (PCCs) between m^6^A-methylated DElncRNAs and DEGs. The false discovery rate (FDR)-adjusted *p*-value was used for multiple testing correction. Pairs with a PCC >0.95 and FDR <0.05 were selected. The lncRNA-mRNA co-expression network was established using Cytoscape (version 3.6.1) (Shannon et al., 2003). The topological properties of this co-expression network, including node degree, betweenness, and closeness, were analyzed using the CytoNCA plugin (version 2.1.6) (Tang et al., 2015).

### Functional enrichment analysis

Gene Ontology (GO) biological process (BP) (The Gene Ontology Consortium, 2019) and Kyoto Encyclopedia of Genes and Genomes (KEGG) (Kanehisa and Goto, 2000) pathway enrichment analyses were performed using the clusterProfiler package (version 3.16.0) to elucidate the functions of m^6^A-methylated DElncRNAs and DEGs in the co-expression network (Yu et al., 2012). A value of *p* < 0.05 (adjusted by the BH method) was considered significant.

### Construction of a protein–protein interaction (PPI) network

Based on the STRING (version 11.0) database (Szklarczyk et al., 2015), the interactions between DEGs co-expressed with DElncRNAs were predicted, and a PPI network was constructed. The species was set as mice, and the PPI score was 0.15 (Yang et al., 2020; Zhao et al., 2022).

### Validation of key DElncRNAs and DEGs using qRT-PCR

qRT-PCR was performed to detect the expression of several identified DElncRNAs and DEGs in the NTD and control groups to validate the reliability of the bioinformatics analysis. The DElncRNAs were Gm5165, Mir100hg, Gm19265, Gm10544, A730017L22Rik, and Malat1. The DEGs included *Zfp236*, *Erc2*, *Nudcd3*, and *Hmg20a*. qRT-PCR was conducted as described in 2.3.

### Statistical analysis

All data are presented as the mean ± standard deviation (SD). The differences between the NTD and control groups were analyzed using Student’s *t*-tests in GraphPad Prism 5.0 (GraphPad Software, San Diego, CA, USA). *p* < 0.05 was considered statistically significant.

## Results

### Overall m^6^A levels and *METTL3* expression were decreased in NTDs

**FIGURE 1:**
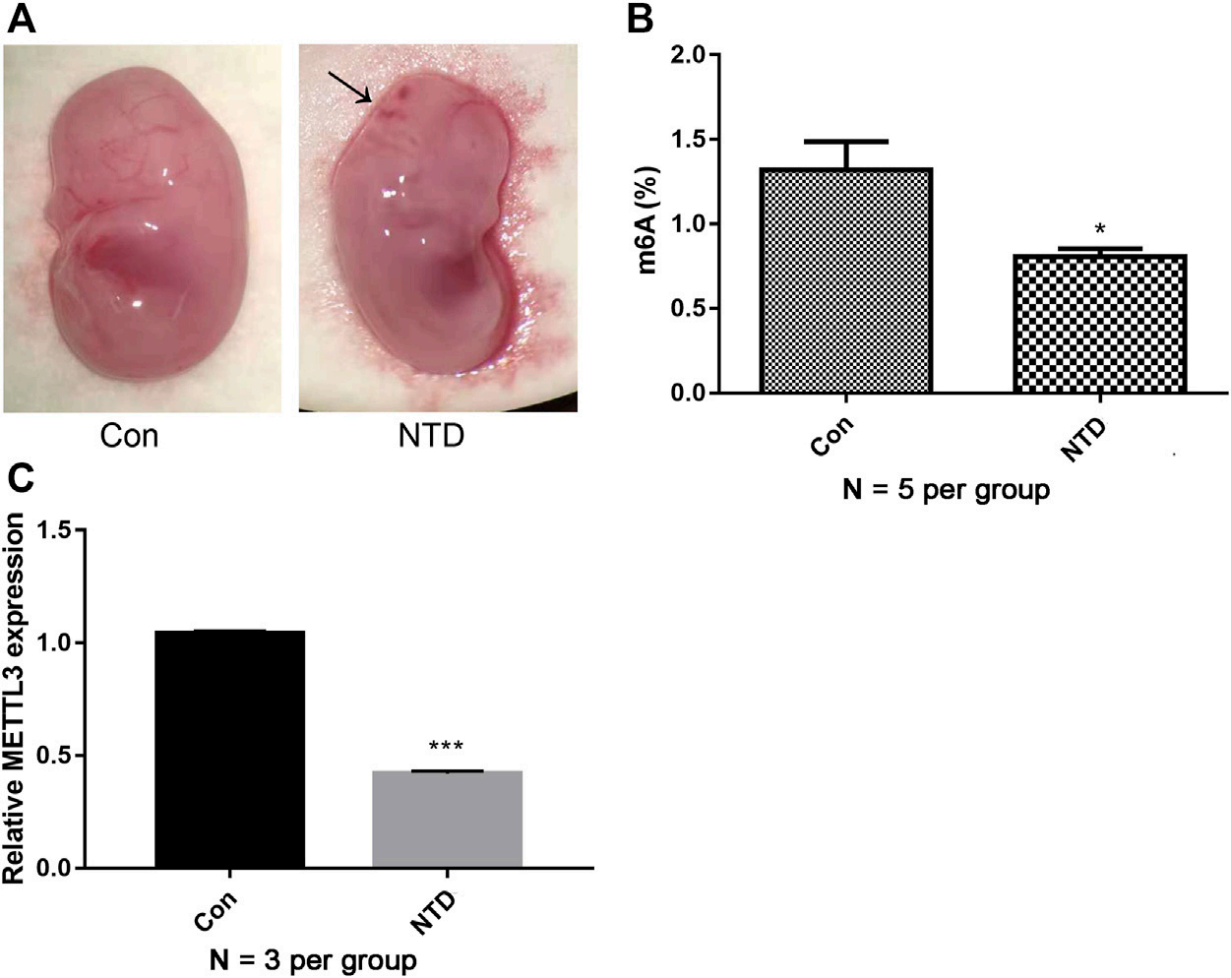
m^6^A levels and *METTL3* expression were decreased in NTDs. **(A)** The dissecting microscope images for embryo in the control group and NTD groups on E16.5d. Arrows indicated the site of defects. **(B)** A quantitative m^6^A analysis was conducted to explore m^6^A enrichment in the brain tissues of NTD and control embryos (N = 5 per group). **(C)** qRT-PCR was carried out to investigate *METTL3* expression in the brain tissues of NTD and control embryos (N = 3 per group). Compared to control group, *p < 0.05 and ***p < 0.001.

We first established NTD mouse models by the intragastric administration of corn oil-dissolved retinoic acid. In the control group, the brains of mouse embryos were completely closed, the appearance was full and smooth, the optic vesicle and otic vesicle were visible, and the spinal surface was intact without laceration. In the NTD group, the rate of stillbirth increased. Some typical morphological malformations were observed, including cracks in the top of the skull, abnormalities of the hindbrain and face, and spina bifida (Figure 1A). These data suggested that the retinoic acid-induced NTD mouse model was successfully established. We then analyzed overall m^6^A levels in NTD and control brain samples. Compared with levels in the control group, the NTD group had significantly lower m^6^A levels, indicating that methylation reactions could be compromised in NTD embryos (Figure 1B). Furthermore, *METTL3* expression in the NTD group was remarkably lower than that in the control group (Figure 1C), indicating that *METTL3* might be essential for m^6^A modification in NTDs.

### Identification of differentially methylated m^6^A sites based on MeRIP-seq data

**FIGURE 2:**
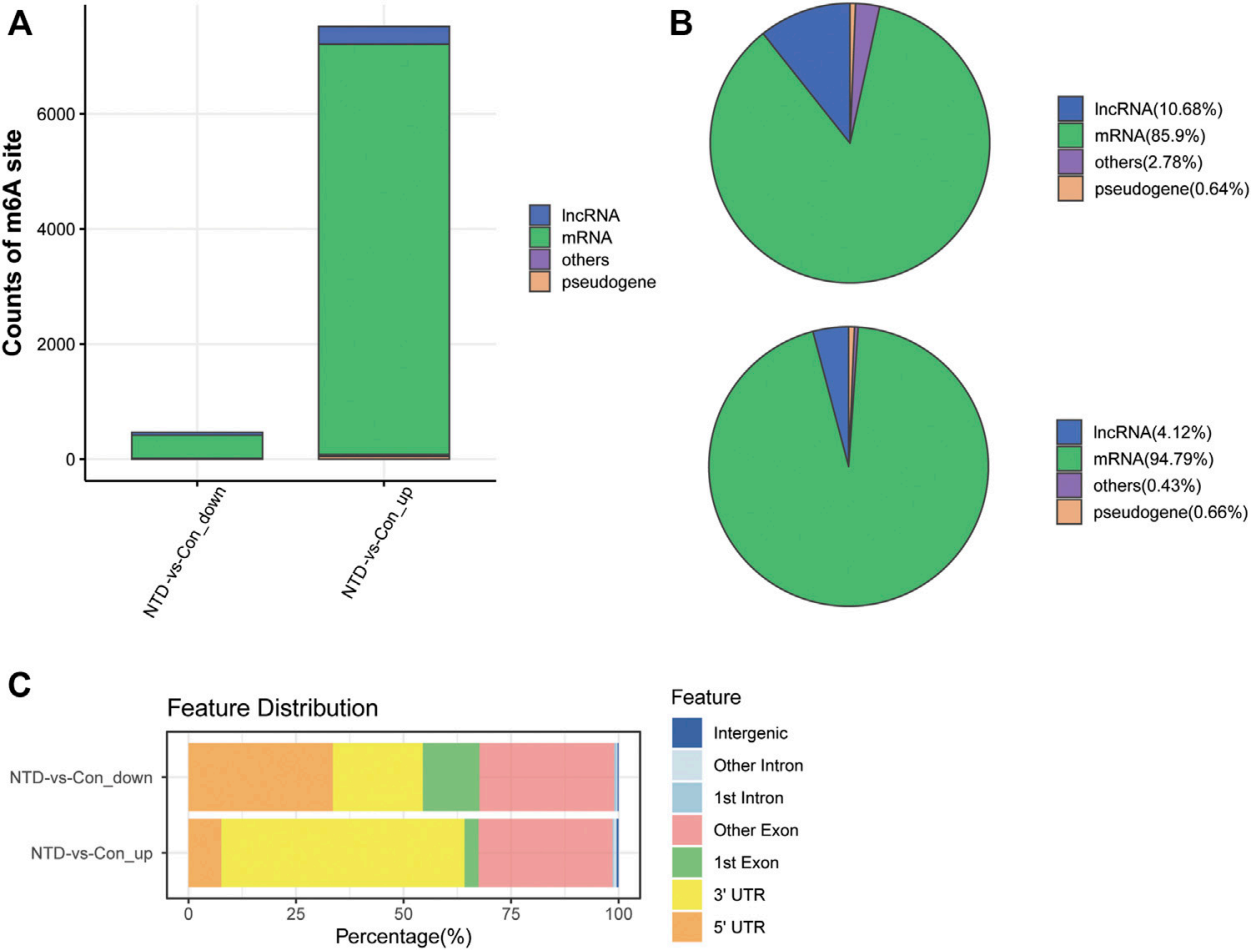
Analysis of differentially methylated m^6^A sites based on MeRIP-seq data. **(A)** Counts of differentially methylated m^6^A sites between the NTD and control groups. **(B)** RNA categories of hypomethylated m^6^A sites. **(C)** RNA categories of hypermethylated m^6^A sites. (D) Distribution of differentially methylated m^6^A sites in functional regions.

The NTD and control groups (N = 3 per group) were subjected to MeRIP-seq. Supplementary Table S1 provides a statistical summary of the raw and clean reads obtained by MeRIP-seq. After sequence alignment to the reference genome, the rates of uniquely mapped reads were higher than 70% (Supplementary Table S2), indicating that the data quality was sufficiently high for subsequent analyses. Based on MeRIP-seq data, 468 hypomethylated m^6^A sites and 7,487 hypermethylated m^6^A sites were found in the NTD group compared to the control group (Figure 2A). According to RNA categories, we found that 50, 402, 3, and 13 hypomethylated m^6^A sites were located in lncRNAs, mRNAs, pseudogenes, and other regions, respectively (Figure 2B), and 310, 7,128, 50, and 32 hypermethylated m^6^A sites were located in lncRNAs, mRNAs, pseudogenes, and other regions, respectively (Figure 2C). Figure 2D shows the distribution of differentially methylated m^6^A sites in functional regions after peak annotation. When the mRNA 3′UTR had an m^6^A modification, 81 and 3,502 mRNAs had hypomethylated and hypermethylated m^6^A sites, respectively.

### Screening DElncRNAs and DEGs based on RNA-seq data

**FIGURE 3:**
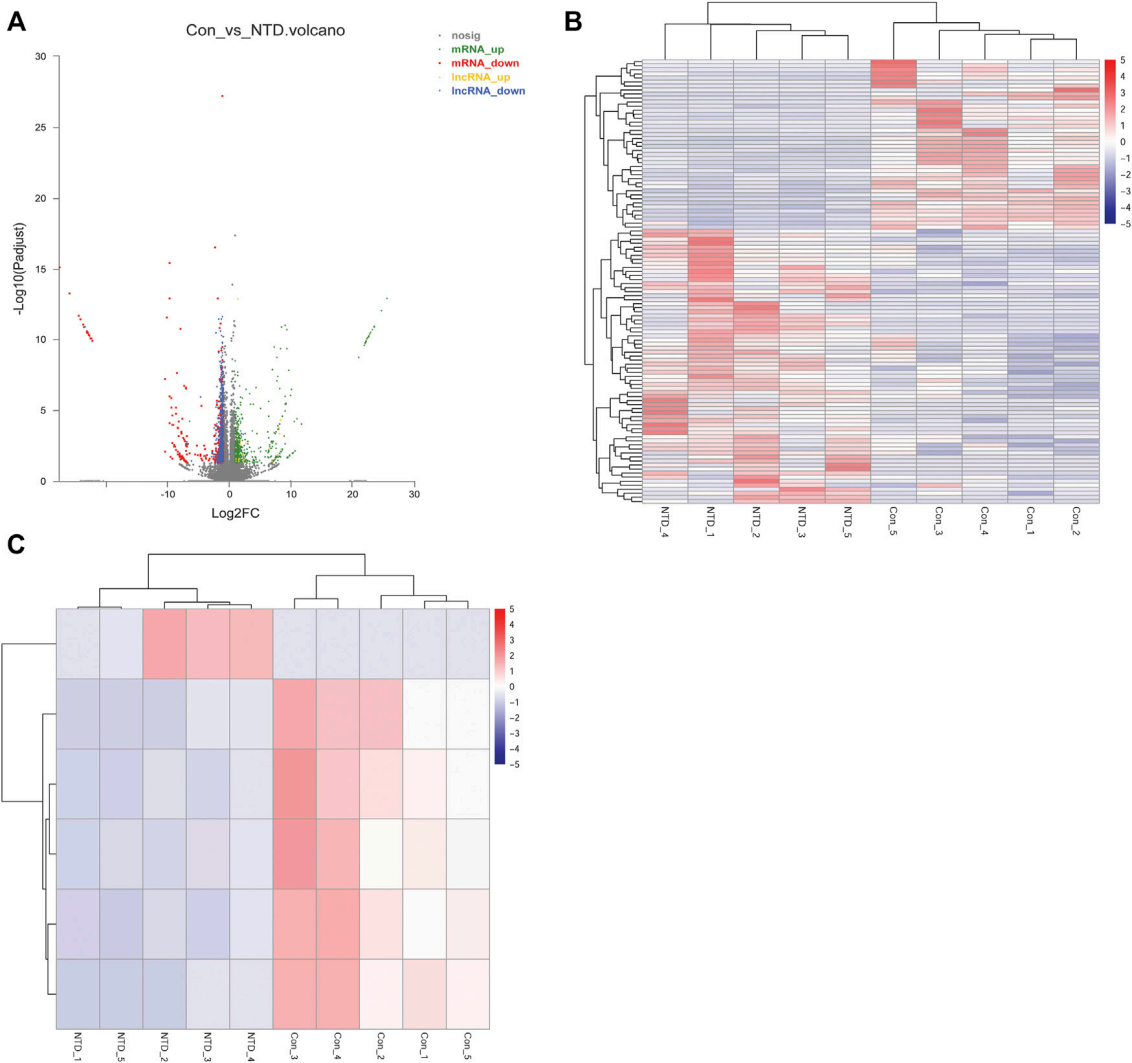
Analysis of DElncRNAs and DEGs between the NTD and control groups based on RNA-seq data. **(A)** Volcano plot of DElncRNAs and DEGs. Green, red, yellow, and blue nodes represent upregulated mRNAs, downregulated mRNAs, upregulated lncRNAs, and downregulated lncRNAs, respectively. **(B)** Heat map of DElncRNAs. **(C)** Heat map of DEGs.

The NTD and control groups (N = 5 per group) were subjected to RNA-seq. In general, 945, 603, 356 raw reads were detected using RNA-seq. Supplementary Table S3 shows a statistical summary of the raw and clean reads obtained from RNA-seq after quality control. After sequence alignment to the reference genome, the rates of uniquely mapped reads were higher than 84.97% (Supplementary Table S4), indicating that the data quality was sufficiently high for subsequent analyses. After a differential expression analysis, 639 DElncRNAs (26 upregulated and 613 downregulated) and 1,132 DEGs (618 upregulated and 514 downregulated) were identified between the NTD and control groups (Figure 3A). As shown in a heatmap, the identified DElncRNAs (Figure 3B) and DEGs (Figure 3C) could distinguish NTD samples from control samples.

### Identification of m^6^A-related DElncRNAs and DEGs

Further integrative analyses of lncRNAs/mRNAs with differentially methylated m^6^A sites and DElncRNAs/DEGs yielded 13 differentially m^6^A-methylated DElncRNAs (corresponding to 20 transcripts) and 170 differentially m^6^A-methylated DEGs (corresponding to 201 transcripts).

### Analysis of lncRNA-mRNA co-expression and PPI networks

**FIGURE 4:**
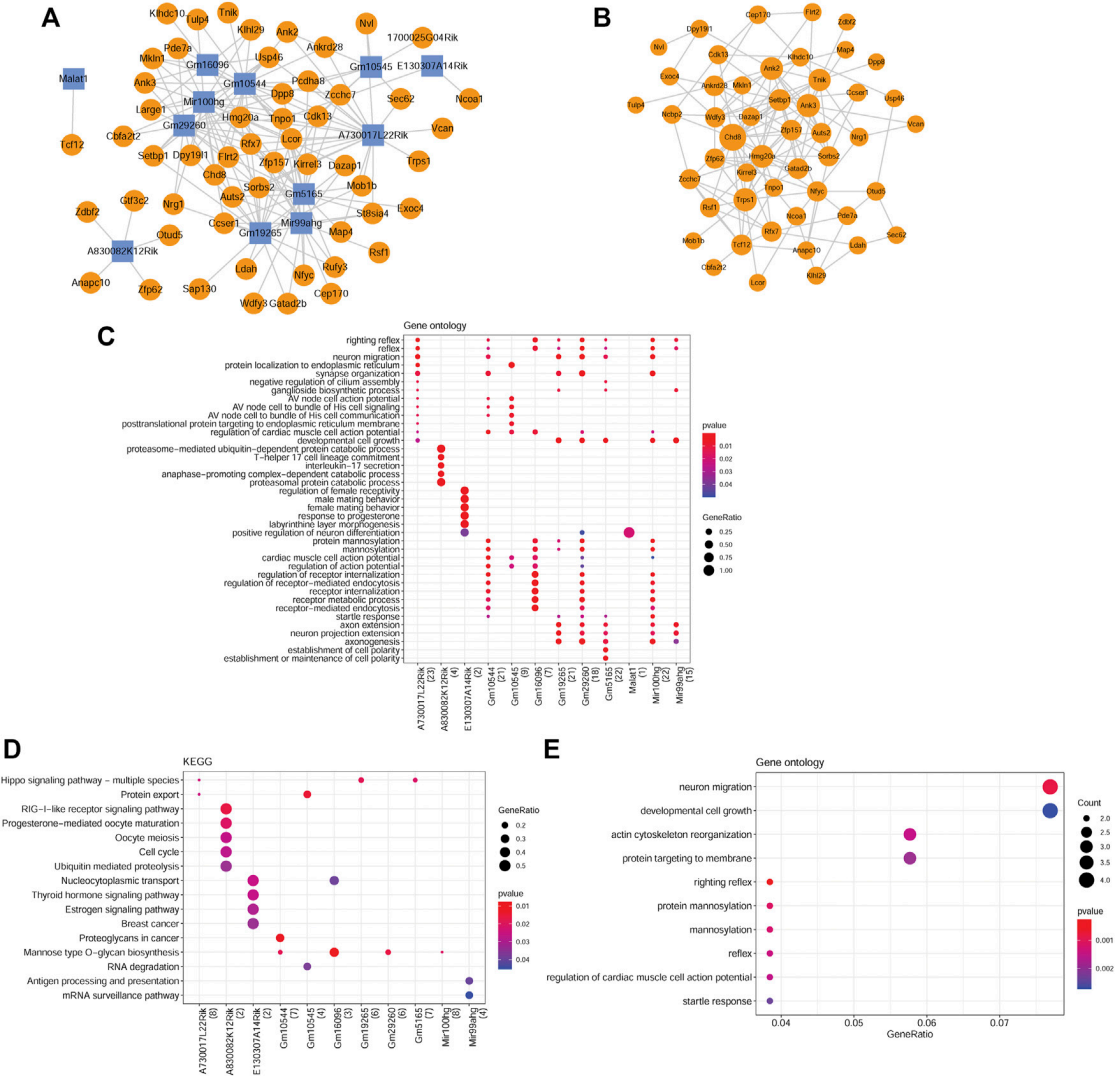
Analysis of co-expressed DElncRNAs and DEGs. **(A)** lncRNA-mRNA co-expression network. Yellow circles represent downregulated mRNAs. Blue squares represent downregulated lncRNAs. **(B)** PPI network constructed by co-expressed DEGs. **(C)** GO-BP terms enriched by co-expressed DElncRNAs. **(D)** KEGG pathways enriched by co-expressed DElncRNAs. **(E)** GO-BP terms enriched by co-expressed DEGs. The size of a node corresponds to the size of the degree.

Based on PCC scores, 171 co-expression pairs were obtained, including nine differentially m^6^A-methylated DElncRNAs and 58 differentially m^6^A-methylated DEGs. Figure 4A shows the co-expression network. By analyzing the topological properties of nodes in the co-expression network, it was observed that lncRNAs, such as Mir100hg, A730017L22Rik, Gm10544, Gm5165, and Gm19265, had higher degrees than those of DEGs. In addition, a PPI network was established based on interactions between differentially m^6^A-methylated DEGs and included 49 nodes and 131 interaction pairs (Figure 4B). All DEGs in the network were downregulated in NTD.

### Functional enrichment analyses of nodes in the lncRNA-mRNA co-expression network

Functional enrichment analyses of the differentially m^6^A-methylated DElncRNAs and DEGs were performed. In total, 1250 GO-BP terms and 23 KEGG pathways were significantly enriched for differentially m^6^A-methylated DElncRNAs. For instance, A730017L22Rik, Gm19265, Gm29260, Gm5165, and Mir100 hg were significantly enriched in developmental cell growth (Figure 4C). Gm10545, Gm16096, Gm29260, and Mir100 hg were involved in mannose-type *O*-glycan biosynthesis. A730017L22Rik, Gm19265, and Gm5165 were involved in the Hippo signaling pathway (Figure 4D). Moreover, the differentially m^6^A-methylated DEGs were associated with 125 GO-BP terms, including neuron migration (Figure 4E), and one KEGG pathway, mannose-type *O*-glycan biosynthesis.

### Validation by qRT-PCR

**FIGURE 5:**
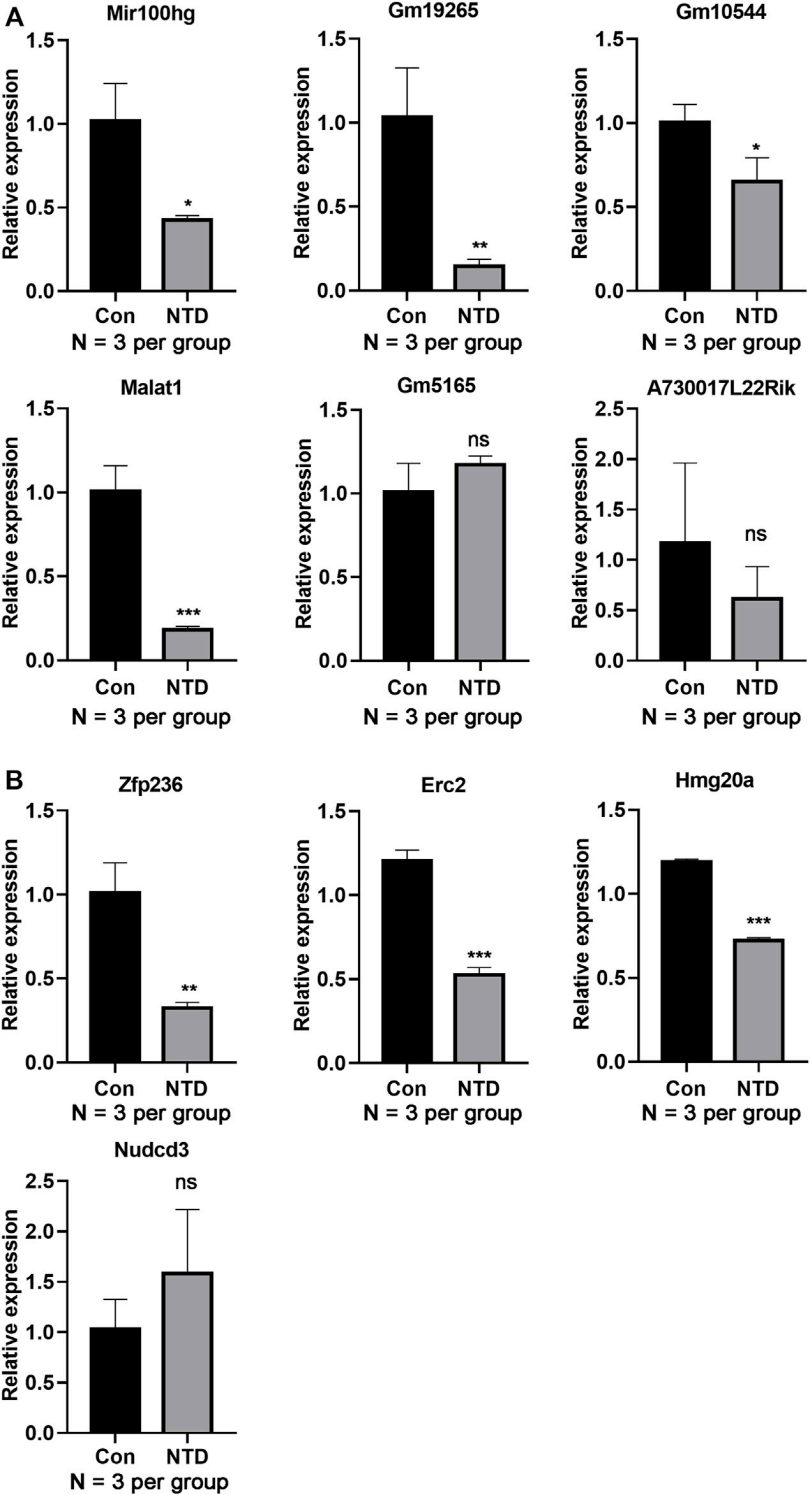
qRT-PCR assay verified the lncRNA and gene expression levels in the brain tissues of NTD and control embryos. **(A)** Expression levels of lncRNAs, such as Mir100hg, Gm19265, Gm10544, and Malat1 (N = 3 per group). **(B)** Expression levels of genes, such as *Zfp236*, *Erc2*, and *Hmg20a* (N = 3 per group). Compared to control group, *p < 0.05, **p < 0.01, and ***p < 0.001.

The expression levels of several DElncRNAs and DEGs were evaluated using qRT-PCR. The NTD group had dramatically lower expression levels of lncRNA, including Mir100hg, Gm19265, Gm10544, and Malat1, than those in the control group (*p* < 0.05, Figure 5A). Similarly, expression levels of key genes, such as *Zfp236*, *Erc2*, and *Hmg20a*, were lower in the NTD group than in the control group (*p* < 0.05, Figure 5B). These data were in line with the results of the bioinformatics analysis. There were no significant differences in Gm5165, A730017L22Rik, or Nudcd3 expression between the NTD and control groups.

## Discussion

m^6^A modifications contribute to the regulation of gene expression during embryonic development (Li C. et al., 2021). *METTL3* has been identified as a critical methyltransferase in m^6^A modification and has functions in various biological processes (Liu S. et al., 2020). The *METTL3*-mediated m^6^A modification is essential for mammalian embryonic development (Sui et al., 2020). A previous study has revealed that levels of m^6^A modification and *METTL3* expression are lower in ethionine-induced NTD than in controls (Zhang L. et al., 2021). Consistent with these findings, we found that the retinoic acid-induced NTD mouse model had dramatically decreased overall m^6^A levels and *METTL3* expression levels than those in control mice. These findings suggested that *METTL3*-mediated m^6^A modifications may be involved in NTD development.

m^6^A modifications can modulate the expression of RNAs, including lncRNAs (Meyer and Jaffrey, 2014). There is increasing evidence that m^6^A-mediated epitranscriptomic changes can modulate lncRNAs in the developing cortex of mouse brains (Nie et al., 2021). We identified differentially m^6^A-methylated DElncRNAs associated with NTDs *via* an integrative analysis of MeRIP-seq and RNA-seq data, including Mir100hg, Gm19265, Gm10544, and Malat1. The neurogenic lncRNA Mir100 hg is a miR-125b and let-7 precursor. Both are implicated in neural development (Rybak et al., 2008; Le et al., 2009). Malat1 is extremely abundant in brain tissues and is associated with neurological disorders, such as stroke, Alzheimer’s disease, and retinal neurodegeneration (Zhang et al., 2017; Meng et al., 2019). In addition to lncRNAs, we identified m^6^A-methylated DEGs, including *Zfp236*, *Erc2*, and *Hmg20a*. *Hmg20a* is mainly expressed in hypothalamic astrocytes and is crucial for neuronal integrity preservation (Lorenzo et al., 2021). *Erc2* participates in synaptic and neuronal functions (Lenihan et al., 2017). Nevertheless, the specific functions of *Gm19265*, *Gm10544*, and *Zfp236* in neuronal development and nervous system-related diseases have not been reported. Our results showed that these lncRNAs and genes are downregulated in NTD, implying that their dysregulation may be associated with NTD development. Further studies are needed to investigate the roles of these lncRNAs and genes in NTDs.

Notably, the differentially m^6^A-methylated DElncRNAs and DEGs were significantly enriched in multiple GO terms and KEGG pathways. *O*-Mannose-linked glycans are highly enriched in the brain and are vital for nervous system functions (Morise et al., 2014). They are also involved in brain development and remyelination (Gao et al., 2019). Our analysis revealed that Gm10545, Gm16096, Gm29260, and Mir100 hg were involved in mannose-type *O*-glycan biosynthesis, providing a mechanism by which m^6^A-methylated DElncRNAs affected the development of NTDs. Moreover, A730017L22Rik, Gm19265, and Gm5165 were implicated in the Hippo signaling pathway, which has established roles in neuronal cell differentiation, neuroinflammation, and neuronal death (Cheng et al., 2020; Li X. et al., 2021). The Hippo/YAP signaling pathway has been implicated in the development of neurological diseases (Bao et al., 2017). Our findings revealed the potential role of the Hippo signaling pathway in NTD development. Moreover, the identified differentially m^6^A-methylated DEGs were significantly enriched in GO-BP terms, including neuron migration, and one KEGG pathway, mannose-type *O*-glycan biosynthesis. Neuronal migration is a fundamental step in nervous system formation (Stockinger et al., 2011). These pathways and GO functions provide insight into the roles of key m^6^A-methylated DElncRNAs and DEGs in the development of NTD.

In conclusion, our study is the first to identify differentially m^6^A-methylated DElncRNAs and DEGs associated with NTDs. Our findings demonstrated that *METTL3*-mediated m^6^A modifications may be involved in the development of NTD. In particular, decreased expression levels of lncRNAs, such as Mir100hg, Gm19265, Gm10544, and Malat1, as well as key genes, such as *Zfp236*, *Erc2*, and *Hmg20a,* may be crucial in NTD development. However, the sample size was small, and the expression levels of the identified m^6^A-methylated lncRNAs and genes were not validated in neural tube cells isolated from fetal brains. Additional functional experiments are needed to reveal the regulatory mechanisms of key DElncRNAs and DEGs in NTDs.

## Data Availability

The raw data of MeRIP-seq and RNA-seq have been deposited in the NCBI Sequence Read Archive (SRA) database under accession number PRJNA879256, respectively.

## References

[B1] Aliperti V. Skonieczna J. Cerase A. (2021). Long non-coding RNA (lncRNA) roles in cell biology, neurodevelopment and neurological disorders. Noncoding. RNA 7, 36. 10.3390/ncrna7020036 34204536 PMC8293397

[B2] Avagliano L. Massa V. George T. M. Qureshy S. Bulfamante G. P. Finnell R. H. (2019). Overview on neural tube defects: From development to physical characteristics. Birth Defects Res. 111, 1455–1467. 10.1002/bdr2.1380 30421543 PMC6511489

[B3] Bao X. He Q. Wang Y. Huang Z. Yuan Z. (2017). The roles and mechanisms of the Hippo/YAP signaling pathway in the nervous system. Yi chuan= Hered. 39, 630–641. 10.16288/j.yczz.17-069 28757477

[B4] Benjamini Y. Hochberg Y. (1995). Controlling the false discovery rate: A practical and powerful approach to multiple testing. J. R. Stat. Soc. Ser. B 57, 289–300. 10.1111/j.2517-6161.1995.tb02031.x

[B5] Bolger A. M. Lohse M. Usadel B. (2014). Trimmomatic: A flexible trimmer for Illumina sequence data. Bioinforma. Oxf. Engl. 30, 2114–2120. 10.1093/bioinformatics/btu170 PMC4103590 24695404

[B6] Cheng J. Wang S. Dong Y. Yuan Z. (2020). The role and regulatory mechanism of Hippo signaling components in the neuronal system. Front. Immunol. 11, 281. 10.3389/fimmu.2020.00281 32140159 PMC7042394

[B7] Cui X. Meng J. Zhang S. Chen Y. Huang Y. (2016). A novel algorithm for calling mRNA m6A peaks by modeling biological variances in MeRIP-seq data. Bioinforma. Oxf. Engl. 32, i378–i385. 10.1093/bioinformatics/btw281 PMC4908365 27307641

[B8] Cui X. Zhang L. Meng J. Rao M. K. Chen Y. Huang Y. (2018). MeTDiff: A novel differential RNA methylation analysis for MeRIP-seq data. IEEE/ACM Trans. Comput. Biol. Bioinform. 15, 526–534. 10.1109/TCBB.2015.2403355 29610101

[B9] Dobin A. Davis C. A. Schlesinger F. Drenkow J. Zaleski C. Jha S. (2013). Star: Ultrafast universal RNA-seq aligner. Bioinforma. Oxf. Engl. 29, 15–21. 10.1093/bioinformatics/bts635 PMC3530905 23104886

[B10] Finnell R. H. Caiaffa C. D. Kim S. E. Lei Y. Steele J. Cao X. (2021). Gene environment interactions in the etiology of neural tube defects. Front. Genet. 12, 659612. 10.3389/fgene.2021.659612 34040637 PMC8143787

[B11] Gao T. Yan J. Liu C.-C. Palma A. S. Guo Z. Xiao M. (2019). Chemoenzymatic synthesis of O-mannose glycans containing sulfated or nonsulfated HNK-1 epitope. J. Am. Chem. Soc. 141, 19351–19359. 10.1021/jacs.9b08964 31738061

[B12] Gong C. Fan Y. Liu J. (2021). METTL14 mediated m6A modification to LncRNA ZFAS1/rab22a: A novel therapeutic target for atherosclerosis. Int. J. Cardiol. 328, 177. 10.1016/j.ijcard.2020.12.002 33301833

[B13] Gu Y. Niu S. Wang Y. Duan L. Pan Y. Tong Z. (2021). DMDRMR-mediated regulation of m6A-modified CDK4 by m6A reader IGF2BP3 drives ccRCC progression. Cancer Res. 81, 923–934. 10.1158/0008-5472.CAN-20-1619 33293428

[B14] Huang W. Huang T. Liu Y. Fu J. Wei X. Liu D. (2021). Nuclear factor I-C disrupts cellular homeostasis between autophagy and apoptosis via miR-200b-Ambra1 in neural tube defects. Cell Death Dis. 13, 17–04473. 10.1038/s41419-021-04473-2 34930914 PMC8688449

[B15] Iyer M. K. Niknafs Y. S. Malik R. Singhal U. Sahu A. Hosono Y. (2015). The landscape of long noncoding RNAs in the human transcriptome. Nat. Genet. 47, 199–208. 10.1038/ng.3192 25599403 PMC4417758

[B16] Jiang X. Liu B. Nie Z. Duan L. Xiong Q. Jin Z. (2021). The role of m6A modification in the biological functions and diseases. Signal Transduct. Target. Ther. 6, 74. 10.1038/s41392-020-00450-x 33611339 PMC7897327

[B17] Kakebeen A. D. Niswander L. (2021). Micronutrient imbalance and common phenotypes in neural tube defects. Genesis 59, e23455. 10.1002/dvg.23455 34665506 PMC8599664

[B18] Kanehisa M. Goto S. (2000). Kegg: Kyoto encyclopedia of genes and genomes. Nucleic Acids Res. 28, 27–30. 10.1093/nar/28.1.27 10592173 PMC102409

[B19] Kim D. Langmead B. Salzberg S. L. (2015). Hisat: A fast spliced aligner with low memory requirements. Nat. Methods 12, 357–360. 10.1038/nmeth.3317 25751142 PMC4655817

[B20] Le M. T. Xie H. Zhou B. Chia P. H. Rizk P. Um M. (2009). MicroRNA-125b promotes neuronal differentiation in human cells by repressing multiple targets. Mol. Cell. Biol. 29, 5290–5305. 10.1128/MCB.01694-08 19635812 PMC2747988

[B21] Lenihan J. A. Saha O. Heimer-McGinn V. Cryan J. F. Feng G. Young P. W. (2017). Decreased anxiety-related behaviour but apparently unperturbed NUMB function in ligand of NUMB protein-X (LNX) 1/2 double knockout mice. Mol. Neurobiol. 54, 8090–8109. 10.1007/s12035-016-0261-0 27889896

[B22] Li B. Dewey C. N. (2011). Rsem: Accurate transcript quantification from RNA-seq data with or without a reference genome. BMC Bioinforma. 12, 323. 10.1186/1471-2105-12-323 PMC3163565 21816040

[B23] Li C. Jiang Z. Hao J. Liu D. Hu H. Gao Y. (2021a). Role of N6-methyl-adenosine modification in mammalian embryonic development. Genet. Mol. Biol. 44, e20200253–e20204685. 10.1590/1678-4685-GMB-2020-0253 33999093 PMC8127566

[B24] Li G. Ma L. He S. Luo R. Wang B. Zhang W. (2022). WTAP-mediated m(6)A modification of lncRNA NORAD promotes intervertebral disc degeneration. Nat. Commun. 13, 1469–28990. 10.1038/s41467-022-28990-6 35304463 PMC8933458

[B25] Li X. Li K. Chen Y. Fang F. (2021b). The role of Hippo signaling pathway in the development of the nervous system. Dev. Neurosci. 43, 263–270. 10.1159/000515633 34350875

[B26] Liu P. Zhang B. Chen Z. He Y. Du Y. Liu Y. (2020a). m(6)A-induced lncRNA MALAT1 aggravates renal fibrogenesis in obstructive nephropathy through the miR-145/FAK pathway. Aging 12, 5280–5299. 10.18632/aging.102950 32203053 PMC7138587

[B27] Liu S. Zhuo L. Wang J. Zhang Q. Li Q. Li G. (2020b). METTL3 plays multiple functions in biological processes. Am. J. Cancer Res. 10, 1631–1646. 32642280 PMC7339281

[B28] Lorenzo P. I. Martin Vazquez E. López-Noriega L. Fuente-Martín E. Mellado-Gil J. M. Franco J. M. (2021). The metabesity factor HMG20A potentiates astrocyte survival and reactive astrogliosis preserving neuronal integrity. Theranostics 11, 6983–7004. 10.7150/thno.57237 34093866 PMC8171100

[B29] Meng C. Yang X. Liu Y. Zhou Y. Rui J. Li S. (2019). Decreased expression of lncRNA Malat1 in rat spinal cord contributes to neuropathic pain by increasing neuron excitability after brachial plexus avulsion. J. Pain Res. 12, 1297–1310. 10.2147/JPR.S195117 31114309 PMC6497903

[B30] Meyer K. D. Jaffrey S. R. (2014). The dynamic epitranscriptome: N6-methyladenosine and gene expression control. Nat. Rev. Mol. Cell Biol. 15, 313–326. 10.1038/nrm3785 24713629 PMC4393108

[B31] Morise J. Kizuka Y. Yabuno K. Tonoyama Y. Hashii N. Kawasaki N. (2014). Structural and biochemical characterization of O-mannose-linked human natural killer-1 glycan expressed on phosphacan in developing mouse brains. Glycobiology 24, 314–324. 10.1093/glycob/cwt116 24352591

[B32] Nie Y. Tian G. G. Zhang L. Lee T. Zhang Z. Li J. (2021). Identifying cortical specific long noncoding RNAs modified by m(6)A RNA methylation in mouse brains. Epigenetics 16, 1260–1276. 10.1080/15592294.2020.1861170 33323036 PMC8813070

[B33] Rybak A. Fuchs H. Smirnova L. Brandt C. Pohl E. E. Nitsch R. (2008). A feedback loop comprising lin-28 and let-7 controls pre-let-7 maturation during neural stem-cell commitment. Nat. Cell Biol. 10, 987–993. 10.1038/ncb1759 18604195

[B34] Shannon P. Markiel A. Ozier O. Baliga N. S. Wang J. T. Ramage D. (2003). Cytoscape: A software environment for integrated models of biomolecular interaction networks. Genome Res. 13, 2498–2504. 10.1101/gr.1239303 14597658 PMC403769

[B35] Stockinger P. Maître J.-L. Heisenberg C.-P. (2011). Defective neuroepithelial cell cohesion affects tangential branchiomotor neuron migration in the zebrafish neural tube. Development 138, 4673–4683. 10.1242/dev.071233 21965614

[B36] Sui X. Hu Y. Ren C. Cao Q. Zhou S. Cao Y. (2020). METTL3-mediated m(6)A is required for murine oocyte maturation and maternal-to-zygotic transition. Cell Cycle 19, 391–404. 10.1080/15384101.2019.1711324 31916488 PMC7100890

[B37] Szklarczyk D. Franceschini A. Wyder S. Forslund K. Heller D. Huerta-Cepas J. (2015). STRING v10: Protein-protein interaction networks, integrated over the tree of life. Nucleic Acids Res. 43, D447–D452. 10.1093/nar/gku1003 25352553 PMC4383874

[B38] Tang Y. Chen K. Song B. Ma J. Wu X. Xu Q. (2021). m6A-Atlas: a comprehensive knowledgebase for unraveling the N6-methyladenosine (m6A) epitranscriptome. Nucleic Acids Res. 49, D134–d143. 10.1093/nar/gkaa692 32821938 PMC7779050

[B39] Tang Y. Li M. Wang J. Pan Y. Wu F. X. (2015). CytoNCA: A cytoscape plugin for centrality analysis and evaluation of protein interaction networks. Biosystems. 127, 67–72. 10.1016/j.biosystems.2014.11.005 25451770

[B40] The Gene Ontology Consortium (2019). The gene Ontology Resource: 20 years and still GOing strong. Nucleic Acids Res. 47, D330–D338. 10.1093/nar/gky1055 30395331 PMC6323945

[B41] Xie S. J. Tao S. Diao L. T. Li P. L. Chen W. C. Zhou Z. G. (2021). Characterization of long non-coding RNAs modified by m(6)A RNA methylation in skeletal myogenesis. Front. Cell Dev. Biol. 9, 762669. 10.3389/fcell.2021.762669 34722547 PMC8548731

[B42] Yadav U. Kumar P. Rai V. (2021). Maternal biomarkers for early prediction of the neural tube defects pregnancies. Birth Defects Res. 113, 589–600. 10.1002/bdr2.1842 33188559

[B43] Yang X. Wu W. Pan Y. Zhou Q. Xu J. Han S. (2020). Immune-related genes in tumor-specific CD4(+) and CD8(+) T cells in colon cancer. BMC Cancer 20, 585–07075. 10.1186/s12885-020-07075-x 32571262 PMC7310260

[B44] Yen Y. P. Chen J. A. (2021). The m(6)A epitranscriptome on neural development and degeneration. J. Biomed. Sci. 28, 40–00734. 10.1186/s12929-021-00734-6 34039354 PMC8157406

[B45] Yu G. Wang L. G. Han Y. He Q. Y. (2012). clusterProfiler: an R package for comparing biological themes among gene clusters. Omics a J. Integr. Biol. 16, 284–287. 10.1089/omi.2011.0118 PMC3339379 22455463

[B46] Yu G. Wang L. G. He Q. Y. (2015). ChIPseeker: An R/bioconductor package for ChIP peak annotation, comparison and visualization. Bioinforma. Oxf. Engl. 31, 2382–2383. 10.1093/bioinformatics/btv145 25765347

[B47] Yu J. Mu J. Guo Q. Yang L. Zhang J. Liu Z. (2017). Transcriptomic profile analysis of mouse neural tube development by RNA-Seq. IUBMB life 69, 706–719. 10.1002/iub.1653 28691208

[B48] Zhang L. Cao R. Li D. Sun Y. Zhang J. Wang X. (2021a). Ethionine-mediated reduction of S-adenosylmethionine is responsible for the neural tube defects in the developing mouse embryo-mediated m6A modification and is involved in neural tube defects via modulating Wnt/β-catenin signaling pathway. Epigenetics Chromatin 14, 52–00426. 10.1186/s13072-021-00426-3 34863249 PMC8645112

[B49] Zhang W. Qian Y. Jia G. (2021b). The detection and functions of RNA modification m(6)A based on m(6)A writers and erasers. J. Biol. Chem. 297, 100973. 10.1016/j.jbc.2021.100973 34280435 PMC8350415

[B50] Zhang X. Hamblin M. H. Yin K. J. (2017). The long noncoding RNA Malat1: Its physiological and pathophysiological functions. RNA Biol. 14, 1705–1714. 10.1080/15476286.2017.1358347 28837398 PMC5731810

[B51] Zhao J. Zhao Y. Ma X. Feng H. Jia L. (2022). Outstanding prognostic value of novel ferroptosis-related genes in chemoresistance osteosarcoma patients. Sci. Rep. 12, 5029–09080. 10.1038/s41598-022-09080-5 35322804 PMC8943205

