# Corrigendum: Identifying key m^6^A-methylated lncRNAs and genes associated with neural tube defects via integrative MeRIP and RNA sequencing analyses

**DOI:** 10.3389/fgene.2023.1198975

**Published:** 2023-07-10

**Authors:** Jing Yang, Jing Xu, Luting Zhang, Yingting Li, Min Chen

**Affiliations:** ^1^ Department of Obstetrics, Affiliated Xiaoshan Hospital, Hangzhou Normal University, Hangzhou, Zhejiang, China; ^2^ Department of Obstetrics and Gynecology, The First Affiliated Hospital of Kunming Medical University, Kunming, Yunnan, China; ^3^ Department of Obstetrics and Gynecology, Department of Fetal Medicine and Prenatal Diagnosis, Key Laboratory for Major Obstetric Diseases of Guangdong Province, The Third Affiliated Hospital of Guangzhou Medical University, Guangzhou, Guangdong, China

**Keywords:** neural tube defects, N6-methyladenosine modification, long non-coding RNA, functional enrichment analysis, methylated RNA immunoprecipitation sequencing

In the published article, there was an error in the **author list**. Author Xu J was erroneously excluded. The corrected author list appears below.

“Yang J^1†^, Xu J^2†^, Zhang L^3^, Li Y^3^, Chen M^3*^

These authors contributed equally to this work”

The **Author Contribution** statement has also been updated to reflect Xu J’s contribution to the study, and appears below:

“JY, JX, and MC designed the study. LZ and YL performed the experiment. JY and LZ analyzed the data. JY, JX, and MC wrote the manuscript. All authors read and approved the final article.”

In the published article, Xu J’s affiliations were erroneously omitted. As well as having **affiliation 3**, they should also have “Department of Obstetrics and Gynecology, The First Affiliated Hospital of Kunming Medical University, Kunming, Yunnan, China*”*.

In the published article, there was an error in the **Funding** statement. The funder “Yunnan Province Science and Technology Program (No. 202101AY070001-121)” was erroneously omitted**.** The correct Funding statement appears below:

“This study was supported by the Guangzhou Science and Technology Program (No. 202102010129), Yunnan Province Science and Technology Program (No. 202101AY070001-121) and the Hangzhou Science and Technology Program (No. 20211231Y121)”.

The authors apologize for these errors and state that they do not change the scientific conclusions of the article in any way. The original article has been updated.

