# Supplementary material for: Identifying key m6A-methylated lncRNAs and genes associated with neural tube defects via integrative MeRIP and RNA sequencing analyses
Source: Front Genet. 2022 Nov 22;13:974357. doi: 10.3389/fgene.2022.974357 (PMC9722945; doi:10.3389/fgene.2022.974357)
Supplement: Supplementary file 1 [file Table1.docx]

**Supplementary Table 1** The statistics results of raw reads and clean reads obtained from MeRIP sequencing

| Groups | Samples | Raw reads | Clean reads | Raw bases (G) | Clean bases (G) | Q20 (%) | Q30 (%) | GC content (%) |
| --- | --- | --- | --- | --- | --- | --- | --- | --- |
| Control | Con1-IP | 126838614 | 122900340 | 19.03 | 17.84 | 98.01 | 93.79 | 49.58 |
|  | Con1-Input | 104025964 | 101161078 | 15.60 | 14.22 | 98.48 | 94.98 | 49.63 |
|  | Con2-IP | 78998710 | 77292268 | 11.85 | 10.91 | 98.52 | 95.18 | 51.77 |
|  | Con2-Input | 93477566 | 89605932 | 14.02 | 11.61 | 98.65 | 95.54 | 49.70 |
|  | Con3-IP | 90057254 | 88036012 | 13.51 | 12.18 | 98.50 | 95.16 | 51.57 |
|  | Con3-Input | 75026056 | 71728962 | 11.25 | 9.16 | 98.78 | 95.89 | 51.49 |
| NTD | NTD1-IP | 68065124 | 66621428 | 10.21 | 9.19 | 98.60 | 95.39 | 51.51 |
|  | NTD1-Input | 79144718 | 75724044 | 11.87 | 9.56 | 98.83 | 95.97 | 50.96 |
|  | NTD2-IP | 76126890 | 74640042 | 11.42 | 10.45 | 98.59 | 95.35 | 50.75 |
|  | NTD2-Input | 90688682 | 86626364 | 13.60 | 10.64 | 98.81 | 95.95 | 52.72 |
|  | NTD3-IP | 74012006 | 72738890 | 11.10 | 10.47 | 98.63 | 95.38 | 51.04 |
|  | NTD3-Input | 85622602 | 82035274 | 12.84 | 9.96 | 98.77 | 95.98 | 56.39 |
