# Supplementary material for: Identifying key m6A-methylated lncRNAs and genes associated with neural tube defects via integrative MeRIP and RNA sequencing analyses
Source: Front Genet. 2022 Nov 22;13:974357. doi: 10.3389/fgene.2022.974357 (PMC9722945; doi:10.3389/fgene.2022.974357)
Supplement: Supplementary file 2 [file Table2.docx]

**Supplementary Table 2** The mapped rate results of sequence alignment based on MeRIP sequencing data

| Groups | Samples | Number of input reads | Average input read length | Uniquely mapped reads number | Uniquely mapped reads (%) |
| --- | --- | --- | --- | --- | --- |
| Control | Con1-IP_R1 | 61450170 | 147 | 58035745 | 94.44% |
|  | Con1-IP_R2 | 61450170 | 143 | 58035997 | 94.44% |
|  | Con1-Input_R1 | 50580539 | 142 | 43690244 | 86.38% |
|  | Con1-Input_R2 | 50580539 | 139 | 43673211 | 86.34% |
|  | Con2-IP_R1 | 38646134 | 142 | 35072125 | 90.75% |
|  | Con2-IP_R2 | 38646134 | 139 | 35156972 | 90.97% |
|  | Con2-Input_R1 | 44802966 | 130 | 37287072 | 83.22% |
|  | Con2-Input_R2 | 44802966 | 128 | 37321596 | 83.30% |
|  | Con3-IP_R1 | 44018006 | 139 | 38865923 | 88.30% |
|  | Con3-IP_R2 | 44018006 | 137 | 38964249 | 88.52% |
|  | Con3-Input_R1 | 35864481 | 129 | 28758787 | 80.19% |
|  | Con3-Input_R2 | 35864481 | 126 | 28744352 | 80.15% |
| NTD | NTD1-IP_R1 | 33310714 | 139 | 28432489 | 85.36% |
|  | NTD1-IP_R2 | 33310714 | 136 | 28470093 | 85.47% |
|  | NTD1-Input_R1 | 37862022 | 127 | 29497810 | 77.91% |
|  | NTD1-Input_R2 | 37862022 | 124 | 29505146 | 77.93% |
|  | NTD2-IP_R1 | 37320021 | 141 | 31146569 | 83.46% |
|  | NTD2-IP_R2 | 37320021 | 138 | 31225259 | 83.67% |
|  | NTD2-Input_R1 | 43313182 | 124 | 32397786 | 74.80% |
|  | NTD2-Input_R2 | 43313182 | 121 | 32387897 | 74.78% |
|  | NTD3-IP_R1 | 36369445 | 145 | 29240148 | 80.40% |
|  | NTD3-IP_R2 | 36369445 | 142 | 29288262 | 80.53% |
|  | NTD3-Input_R1 | 41017637 | 123 | 28975529 | 70.64% |

R1 indicates left-end data and R2 indicates right-end data.
