# Supplementary material for: Identifying key m6A-methylated lncRNAs and genes associated with neural tube defects via integrative MeRIP and RNA sequencing analyses
Source: Front Genet. 2022 Nov 22;13:974357. doi: 10.3389/fgene.2022.974357 (PMC9722945; doi:10.3389/fgene.2022.974357)
Supplement: Supplementary file 3 [file Table3.docx]

**Supplementary Table 3** The statistics results of raw reads and clean reads obtained from RNA sequencing

| Groups | Samples | Raw reads | Raw bases | Clean reads | Clean bases | Q20 (%) | Q30 (%) | GC content (%) |
| --- | --- | --- | --- | --- | --- | --- | --- | --- |
| Control | Con1 | 94661078 | 14293822778 | 94037522 | 11608610526 | 98.34 | 95.09 | 47.87 |
|  | Con2 | 113755508 | 17177081708 | 112966806 | 13828295608 | 98.3 | 94.97 | 47.41 |
|  | Con3 | 99302020 | 14994605020 | 98653702 | 12590214006 | 98.24 | 94.83 | 47.6 |
|  | Con4 | 80011370 | 12081716870 | 79484808 | 9887109616 | 98.27 | 94.89 | 47.96 |
|  | Con5 | 91649290 | 13839042790 | 91114532 | 11310980657 | 98.35 | 95.06 | 47.86 |
| NTD | NTD1 | 100885668 | 15233735868 | 100110830 | 12671841056 | 98.23 | 94.92 | 49.78 |
|  | NTD2 | 91629708 | 13836085908 | 90984588 | 11251366967 | 98.2 | 94.85 | 49.72 |
|  | NTD3 | 89939916 | 13580927316 | 89346990 | 11119503587 | 98.22 | 94.88 | 50.26 |
|  | NTD4 | 93104112 | 14058720912 | 92527992 | 11293295570 | 98.34 | 95.05 | 48.2 |
|  | NTD5 | 90664686 | 13690367586 | 89994176 | 11137797224 | 98.23 | 94.9 | 49.87 |
