# Supplementary material for: Identifying key m6A-methylated lncRNAs and genes associated with neural tube defects via integrative MeRIP and RNA sequencing analyses
Source: Front Genet. 2022 Nov 22;13:974357. doi: 10.3389/fgene.2022.974357 (PMC9722945; doi:10.3389/fgene.2022.974357)
Supplement: Supplementary file 4 [file Table4.docx]

**Supplementary Table 4** The mapped rate results of sequence alignment based on RNA sequencing data

| Groups | Samples | Total reads | Total mapped | Multiple mapped | Uniquely mapped |
| --- | --- | --- | --- | --- | --- |
| Control | Con1 | 94037522 | 89780244 (95.47%) | 6696619 (7.12%) | 83083625 (88.35%) |
|  | Con2 | 112966806 | 108397969 (95.96%) | 6872348 (6.08%) | 101525621 (89.87%) |
|  | Con3 | 98653702 | 94318303 (95.61%) | 5428646 (5.5%) | 88889657 (90.1%) |
|  | Con4 | 79484808 | 76054306 (95.68%) | 4448483 (5.6%) | 71605823 (90.09%) |
|  | Con5 | 91114532 | 87459752 (95.99%) | 5146128 (5.65%) | 82313624 (90.34%) |
| NTD | NTD1 | 100110830 | 94853717 (94.75%) | 8143650 (8.13%) | 86710067 (86.61%) |
|  | NTD2 | 90984588 | 85773050 (94.27%) | 7277370 (8.0%) | 78495680 (86.27%) |
|  | NTD3 | 89346990 | 84251623 (94.3%) | 8333807 (9.33%) | 75917816 (84.97%) |
|  | NTD4 | 92527992 | 88568583 (95.72%) | 6788455 (7.34%) | 81780128 (88.38%) |
|  | NTD5 | 89994176 | 85070525 (94.53%) | 7859445 (8.73%) | 77211080 (85.8%) |
